# Concentration-dependent *rhombitrihexagonal tiling* patterns at the liquid/solid interface†
†Electronic supplementary information (ESI) available: With additional STM images, optimized geometries of **1** and **2**. See DOI: 10.1039/c5sc00811e



**DOI:** 10.1039/c5sc00811e

**Published:** 2015-07-22

**Authors:** Vladimir Stepanenko, Ramesh Kandanelli, Shinobu Uemura, Frank Würthner, Gustavo Fernández

**Affiliations:** a Institut für Organische Chemie and Center for Nanosystems Chemistry , Universität Würzburg Am Hubland , 97074 Würzburg , Germany . Email: wuerthner@chemie.uni-wuerzburg.de ; Email: gustavo.fernandez@uni-wuerzburg.de; b Department of Advanced Materials Science , Kagawa Universtity , 2217-20, Hayashi-cho , Takamatsu , Kagawa 761-0396 , Japan

## Abstract

We report STM investigations on a linear oligophenyleneethylene (OPE)-based self-assembling Pd(ii) complex **1** that forms highly-ordered concentration dependent patterns on HOPG. At high concentration, 2D lamellar structures are observed whereas the dilution of the system below a critical concentration leads to the formation of visually attractive rhombitrihexagonal *Archimedean tiling* arrangements featuring three different kinds of polygons: triangles, hexagons and rhombi. The key participation of the Cl ligands attached to the Pd(ii) centre in multiple C–H···Cl interactions was demonstrated by comparing the patterns of **1** with those of an analogous non-metallic system **2**.

## Abstract 2

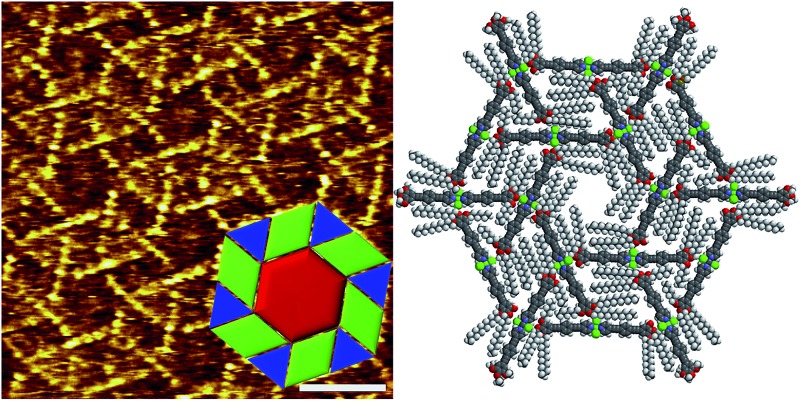
A self-assembling Pd(ii) complex forms sophisticated concentration-dependent *Archimedean tiling* patterns composed of three types of polygons at the liquid/solid interface.

## 

From bee honeycombs to ancient Roman mosaics and Moorish wall tilings, periodic polygonal patterns are ubiquitous in arts and science both for ornamental and technological purposes.^1^ Tessellations of surfaces, *i.e.*, the tiling of planes using one or various types of regular polygons, were first classified by Kepler in 1619.^2^ *Regular tilings* feature a single type of polygon (triangle, square or hexagon) repeated infinitely whereas less frequent *semiregular Archimedean tilings* (AT) combine at least two different polygons placed edge-to-edge around a vertex. Scanning tunnelling microscopy (STM) provides a perfect tool to observe such patterns at the molecular level^3^ and furthermore constitutes a link to nanodevice technology.^4^ For instance, and besides various 1D patterns and/or 2D networks based on a wide variety of self-assembled systems,^5,6^ AT arrangements have been visualized by STM. These systems, however, have been limited to trihexagonal (Kagomé) tilings,^6^ in which two hexagons and two triangles are alternated on each vertex. Very recently, lanthanide-based polyphenyl systems have been demonstrated to form exciting snub square tilings combining two different (trigonal and square) polygons on Ag(111) through STM.^7^

In this article, we move a step further towards complex tessellations at the liquid/solid interface by realizing a special type of rhombitrihexagonal AT patterns that feature up to three different polygons (triangle, rhombus and hexagon). Our system not only represents one of the most complex patterns ever visualized by STM but it can also be transformed into lamellar structures above a critical concentration.^8^

To achieve this goal, we have taken advantage of recent findings from our group on a self-aggregating oligophenyleneethylene (OPE)^9^-based Pd(ii) derivative **1** (Fig. 1).^10^ Complex **1** exists in a monomeric state in nonpolar solvents below ≈1 × 10^–4^ M. Above this critical concentration, micrometre-sized elongated supramolecular structures are formed, in which the OPE-based ligands of **1** adopt a more rigid conformation around the Pd(ii) ion to maximize π–π and Pd···Pd interactions with neighbouring units in the stack.

**Fig. 1:**
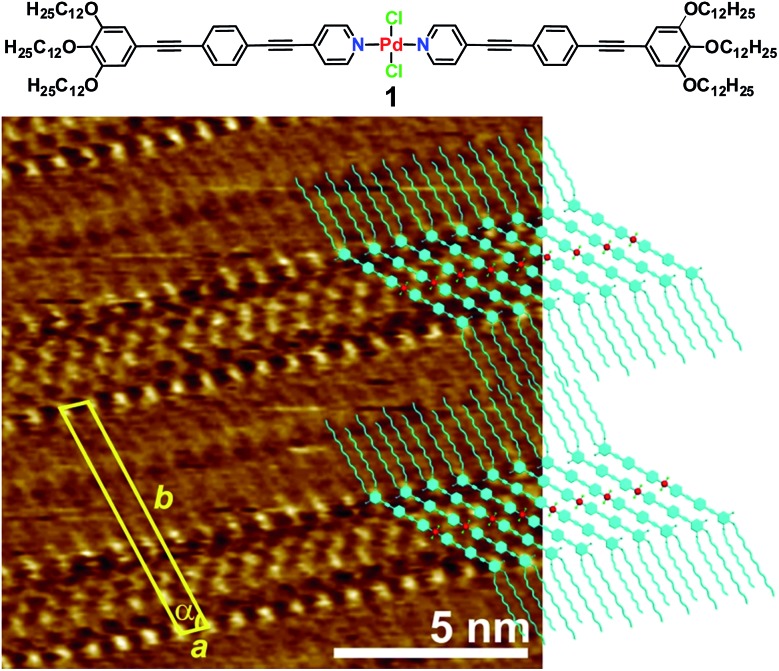
High-resolution STM image of the OPE-based Pd(ii) complex **1** (*c* = 5 × 10^–4^ M) at the HOPG/1-phenyloctane interface and molecular packing within lamellar structures. Tunnelling condition: *V*_bias_ = 750 mV, *I*_set_ = 3.0 pA.

Encouraged by these results, we questioned whether the distinct conformation of the units of **1** in solution depending on the concentration would also be reflected in a different packing mode at the liquid/solid interface, as observed for other metallosupramolecular π-stacks.^11^ To that end, a drop of a concentrated solution of **1** (*c* = 5 × 10^–4^ M) in 1-phenyloctane was placed onto HOPG and investigated by STM. The images show the formation of highly ordered lamellar structures consisting of alternated bright and dark fringes (Fig. 1 and S1†). In contrast, the pyridine-based ligand (precursor of **1**) alone does not form any organized structures on HOPG even at millimolar concentration, thus revealing the importance of an extended aromatic surface and the presence of a Cl–Pd(ii)–Cl fragment to produce organized ad-layers. Within the lamellar structures, the spots with higher local density of states can be assigned to individual aromatic rings belonging to the OPE segments of **1** whereas the darker striations attached to the edges of the aromatic system correspond to parallel-aligned dodecyl chains (Fig. 1). The unit cell of this pattern is highlighted in yellow in Fig. 1 and the corresponding parameters are: *a* = 0.76 ± 0.02 nm, *b* = 6.81 ± 0.06 nm, and *α* = 75.0 ± 3.0°. On the basis of these dimensions, only four out of six dodecyl chains from each molecule (two outer and two inner) are adsorbed on the substrate whereas the remaining two chains are most likely embedded in the supernatant.^12^ The density of the lamellar pattern was calculated to be 0.20 molecules per m^2^ (plane group *p*1).^13^ Remarkably, the parallel orientation of the OPE units at the HOPG/1-phenyloctane interface closely resembles that observed in the associates in nonpolar solutions, although one has to note that in the former assemblies the aromatic rings lie on the HOPG surface whereas in solution these are stacked on top of each other. These observations infer that monolayer formation is largely driven by adsorbate-substrate (epitaxial) and adsorbate–solvent (solubility) interactions.^14^

Similarly to the solution behaviour, we questioned whether dilution of the system below a given concentration (1 × 10^–4^ M) would lead to a distinct molecular arrangement at the liquid/solid interface. Fig. 2 shows the STM images of **1** obtained from a 100-fold more diluted solution (*c* = 1 × 10^–6^ M) of **1** at the HOPG/1-phenyloctane interface. Interestingly, no sign of lamellar structures was observed at this concentration. However and to our surprise, a highly-ordered periodic pattern comprising three types of polygons (hexagons, rhombi and triangles) can be visualized (Fig. 2, S3 and S4†).^15^ These results bring to light that the structural phase transition in solution and at the solid/liquid interface occurs in a similar concentration range (below 1 × 10^–4^ M). On closer scrutiny, we noticed that the geometric shapes are separated from one another by bright segments that correspond to the aromatic rings of the molecules of **1** (Fig. 2a and b). Within this arrangement, each hexagon is sharing its edges with 6 rhombi and its vertices with 6 triangles leaving no gaps and overlaps, as shown in Fig. 2b and c. This exotic surface tessellation (plane group *p*6) resembles one of the AT of the Euclidean plane, the 3.4.6.4. rhombitrihexagonal tiling.^2^ The unique difference from the regular rhombitrihexagonal tiling is the presence of rhombi instead of squares. The density of the AT patterns corresponds to 0.16 molecules per m^2^. By careful analysis of the STM images, we found out that the edges of all polygons are nearly equivalent in length (1.7 ± 0.2 nm) (Fig. 2b). This distance matches that of the aromatic backbone of a pyridine-substituted OPE ligand obtained by theoretical calculations (Fig. S10a†). Indeed, some individual aromatic rings can be distinguished in the magnification shown in Fig. 2b. According to our STM investigations, the Pd(ii) centres are located at the vertices of the polygons, as all edges are occupied by the aromatic segments (see model in Fig. 2c). The repeat unit is represented by an equilateral triangular motif consisting of three molecules, whose edges are successively oriented towards the Cl–Pd(ii)–Cl centre of a neighbouring unit within the triangle, as shown in Fig. 2c. Six such triangular subunits further pack into a hexagonal motif, thereby delineating an inner hexagonal cavity surrounded by six triangles and six rhombi in an alternated fashion (Fig. 2b and c). The dimensions of the unit cell are *a* = 6.5 ± 0.1 nm, *b* = 6.5 ± 0.1 nm, and *α* = 60 ± 3° whereas the distance between the Pd centres within each triangular motif extracted from STM measurements was found to be 2.5 ± 0.2 Å. According to this dimension, the edge of one molecule and the Cl–Pd(ii)–Cl fragment of a neighbouring unit are distant enough to enable the interaction between the central OCH_2_ group of one molecule and the Cl ligand of the other one by C–H···Cl interactions (see proposed model in Fig. 2c and S5, S6†).

**Fig. 2:**
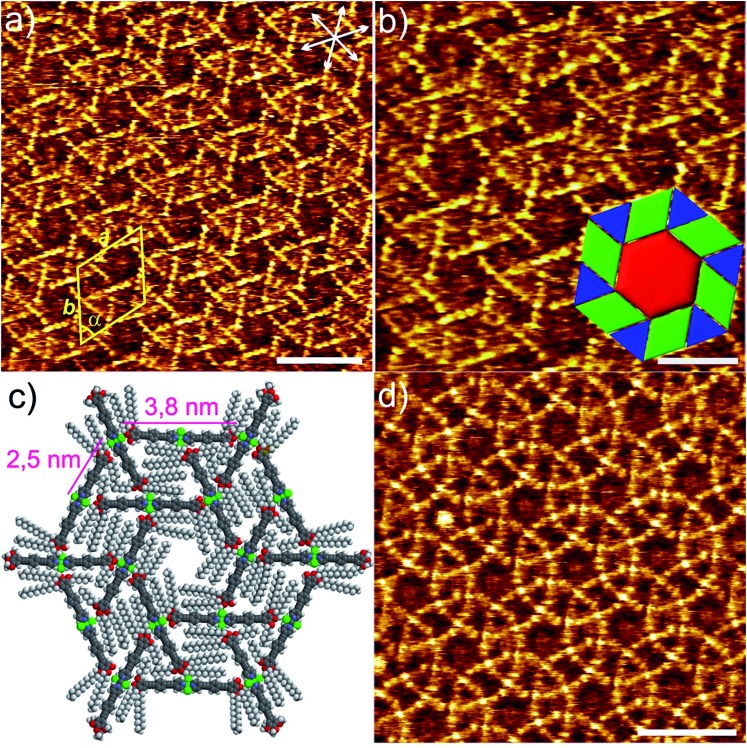
(a, b and d) STM height images of **1** at an HOPG/1-phenyloctane interface (*c* = 1 × 10^–6^ M). *Z* scale is 0.2 nm. Tunnelling condition: *V*_bias_ = 690 mV, *I*_set_ = 8.0 pA. The bar scale in (a and d) corresponds to 8 nm and in (b) to 5 nm. (c) Tentative space-filling model (AM1 level, Spartan) of the AT patterns showing CH···Cl interactions.

As shown by us^16^ and others,^17^ metal-bound chlorine atoms have a strong propensity to interact with polarized C–H groups through hydrogen bonding interactions both in the crystalline state^18^ and in solution.^16^ In our system, only the methylene groups attached to the electronegative oxygen heteroatoms of the side chains are polarized enough to interact with such hydrogen-bonding Cl acceptors. Thus, on the basis of these considerations, STM analysis and theoretical calculations, two C–H···Cl interactions on either side of every molecule of **1** represent, along with the interaction of aromatic and aliphatic segments with the HOPG lattice,^19^ the driving force for AT formation. It is worth noting that due to their lower tunnelling efficiency, a clear visualization of the dodecyl chains has not been possible.^20^ We hypothesize that the alkyl chains will be concentrated in all polygonal cavities to maximize their interaction with the HOPG surface, which ultimately facilitates the AT formation. This situation is clearly possible in the hexagonal cavities, in which up to 12 dodecyl chains can be accommodated, two per monomeric unit (Fig. 2c). The cavities of the rhombi are slightly smaller and we postulate that four chains (see Fig. 2c and S5†) can occupy these areas. Finally, the relatively high electron density observed in the triangular voids suggests that these areas are also considerably filled with alkyl chains.

However, due to their smaller size compared to the rhomboidal and hexagonal cavities only partial adsorption of the chains is possible, whereas other parts protrude above the adsorbate into the phenyloctane layer.^21^ Our proposed model (See Fig. 2c, S5 and S6†) clearly shows that a maximum of 6–7 carbon atoms from each dodecyl chain fit in the triangular voids without inducing severe steric effects or distortions in the AT arrangement.

As particularly apparent in Fig. 2a and b, the majority of the molecules feature a nearly perfect linear geometry, indicating that the pyridine-based ligands are arranged with a 180° angle around the Pd(ii) ion. There are, however, some areas in which some slightly bent molecules can be observed. This is evident in Fig. 2d and S4† top, in which a small distortion of the ideal 180° angle is observed in few molecules resulting in a ring-like appearance. We also observed that this bending is not periodic but rather randomly distributed over the whole HOPG surface. The phenomenon of molecular curvature of systems exhibiting an extended π-conjugated surface has been previously observed for different classes of molecules.^22^ In a particularly relevant example, Beton, Anderson and co-workers have recently reported on a novel 2D supramolecular organization of cyclic porphyrin systems by STM.^23^ They describe the encapsulation of one cyclic polymer in a folded state into another unfolded polymer. The folded polymer undergoes bending where the subsequent strain is adequately made up by stacking stability. Accordingly, the bending energy of the complex in the AT patterns was calculated using the following equation:*E* = 0.5*Kl*/*R* ^2^where *K* is the bending coefficient, *l* is the length of the molecule and *R* is radius of curvature in the molecular arrangement. We applied this equation to calculate the bending energy of our **1** taking into account that the bending coefficient corresponds to 0.03 nN nm^2^ (for monolayer systems), the molecular length is 3.8 nm and *R* can be approximated to 4 nm. The relatively small estimated energy (3.56 meV) required for the bending around the metal ion is well compensated by the high stability of the multipolygonal tiling that is attributed to C–H···Cl interactions and alkyl chain packing.

In order to find out to what extent the existence of a Cl–Pd(ii)–Cl fragment and thus, the participation of C–H···Cl forces is influencing the AT formation, we have investigated a non-metallic OPE-based analogue **2** through STM. OPE **2** (Fig. 3)^10^ is equivalent in size to complex **1** (see geometry-optimized structures in Fig. S11†). However, the Cl–Pd(ii)–Cl fragment has now been replaced by an alkyne functionality. This slight modification is expected to prevent C–H···Cl interactions and, consequently, the formation of multipolygonal patterns. Similarly to **1**, OPE **2** forms one-dimensional associates in nonpolar solvents above 1 × 10^–4^ M, although the propensity of this system to aggregate is considerably reduced compared to 1.^10^ In fact, when a 5 × 10^–4^ M solution of **2** in phenyloctane was used for STM under equivalent conditions to those of **1**, no lamellar patterns were observed, highlighting again that the solution and interface behaviour are comparable. Moreover, dilution of the sample up to 10^–6^ M did not lead to any changes in the molecular packing on HOPG, as shown in Fig. 3 and S8.† Regardless of the concentration, a highly regular grid-like pattern consisting of bright segments of 4.0 ± 0.2 nm in length is observed (Fig. 3a and b). The good agreement between this length and that extracted from molecular modelling (3.83 nm, Fig. 3c and S11†) supports that these fragments correspond to the aromatic OPE core of **2**.

**Fig. 3:**
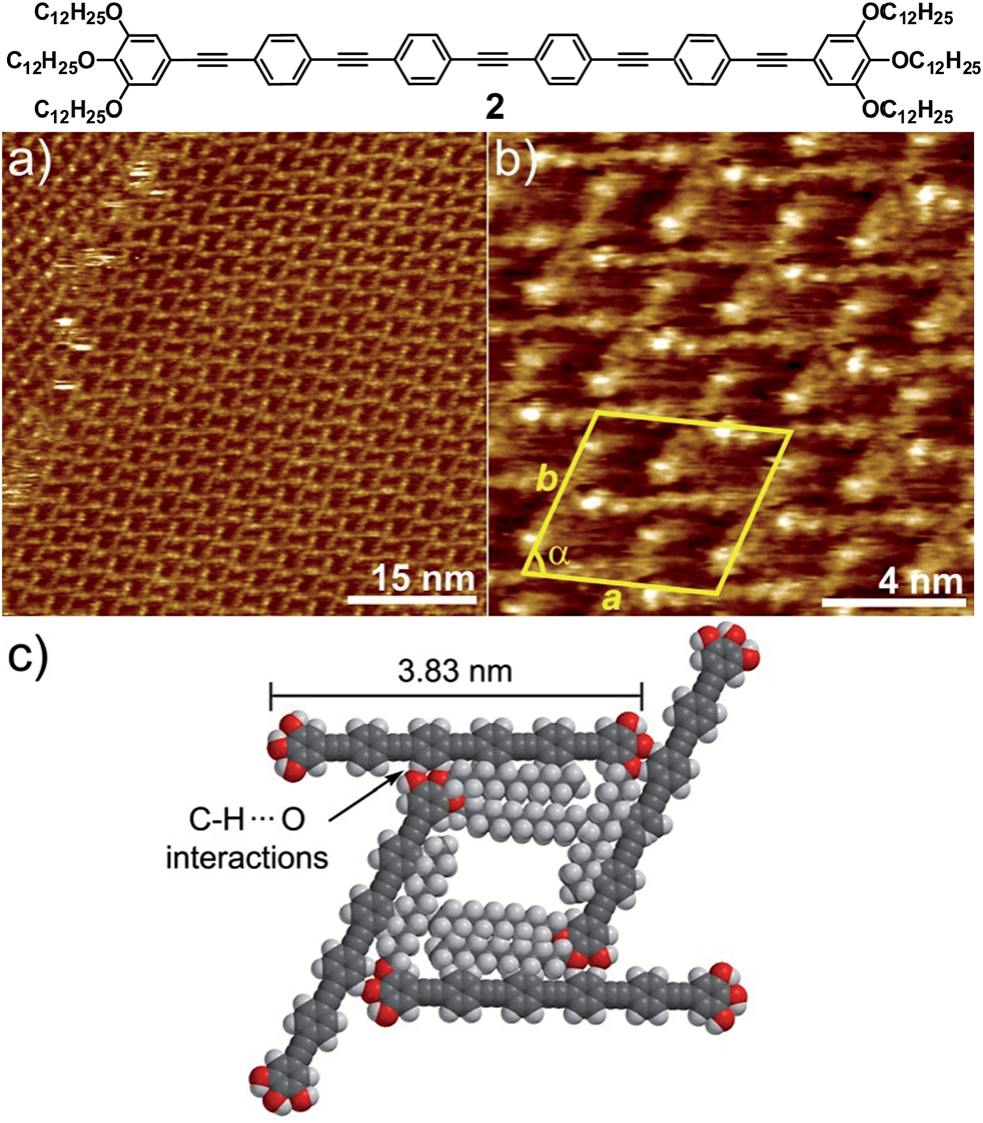
(a and b) STM height images of **2** at an HOPG/1-phenyloctane interface (*c* = 1 × 10^–6^ M). *Z* scale is 0.2 nm. Tunnelling condition: *V*_bias_ = 690 mV, *I*_set_ = 8.0 pA; (c) proposed molecular packing of **2** within grid-like patterns in which all alkyl chains are on the HOPG surface showing C–H (aromatic)···O interactions.

In contrast to **1**, the absence of a relatively flexible Pd(ii) centre increases the rigidity of the system to the point that bending of the molecules cannot be realized. Similarly to **1**, the alkyl chains have not been visualized and are most likely adsorbed onto HOPG occupying the empty spaces between the OPE segments, as depicted in the model shown in Fig. 3c and S9.† The repeat unit comprises four molecules that delimit a cavity with a quadrilateral shape (Fig. 3b), yielding a unit cell whose parameters are *a* = 5.2 ± 0.2 nm, *b* = 5.2 ± 0.2 nm, and *α* = 74.0 ± 3.0° (plane group *p*4). However and in contrast to **1**, no rhombitrihexagonal structures are formed. This is influenced by the absence of chlorine ligands that can participate in weak C–H···Cl hydrogen bonding interactions with polarized CH_2_ groups. As a result, the grid-like pattern formed by **2** should be stabilized by other weak interactions. According to the proposed model shown in Fig. 3c, the patterns are maintained by weak CH···O forces between the hydrogens of the aromatic rings connected to the central triple bond and the oxygen atoms of the dodecyloxy chains. On this basis, each molecule interacts with four neighboring molecules through a total number of eight C–H···O contacts: four of them involving four of the oxygen atoms of the peripheral chains and the remaining four involving the two central aromatic rings, two on each side, thus creating a uniform structure exhibiting a network density of 0.15 molecules per nm^2^.

## Conclusions

In summary, we have observed distinct patterns through STM by exploiting the self-assembly behaviour of an OPE-based Pd(ii) complex **1**. Above a critical concentration, the units of **1** are arranged in a parallel fashion into lamellar patterns. In more diluted solutions, however, the involvement of the Cl–Pd(ii)–Cl fragment of **1** in C–H···Cl interactions with oxygen-polarized CH_2_ groups of the side chains along with surface effects of the HOPG lattice lead to one of the most complex tessellations ever visualized by STM: a special type of semiregular rhombitrihexagonal tiling. The key influence of the Cl–Pd(ii)–Cl center is demonstrated by investigating a related non-metallic compound **2**. Our findings bring to light that unconventional non-covalent forces such as C–H···X interactions may become relevant enough to strongly influence pattern formation. Such surface tessellations with uniform porosity may be exploited for the encapsulation of guest molecules on surfaces, providing access to surface-active 2D or 3D assemblies, as recently shown by Tait, Flood and co-workers.^24^
