# Supplementary material for: Concentration-dependent rhombitrihexagonal tiling patterns at the liquid/solid interface
Source: Chem Sci. 2015 Jul 22;6(10):5853–8. doi: 10.1039/c5sc00811e (PMC5523081; doi:10.1039/c5sc00811e)
Supplement: Supplementary file 1 [file SC-006-C5SC00811E-s001.pdf]

Electronic Supplementary Material (ESI) for Chemical Science.
This journal is © The Royal Society of Chemistry 2015

Supporting Information

Concentration-Dependent Rhombitrihexagonal Tiling patterns at the Liquid-Solid Interface

Vladimir Stepanenko, Ramesh Kandanelli, Shinobu Uemura, Frank Würthner* and Gustavo Fernández*

1. **Details of STM Instrumentation and sample preparation** **2**
2. **STM images of 1 showing lamellar patterns** **3, 4**
3. **STM images of 1 showing *Tiling* patterns** **4, 5**
4. **Computed Models of the AT Patterns** **6,7**
5. **STM image of 1 showing both lamellar and Tilling patterns** **7**
6. **STM images of 2 and computed model of the grid-like Patterns** **8, 9**
7. **Geometry-optimized Structures of 1 and 2** **10**

STM Instrumentation

STM measurements were performed with a commercial Bruker AXS MultiMode^{TM}Nanoscope IV scanning probe microscope using mechanically cut Pt/Ir (90:10) tips (MakTecK GmbH, Germany).

Sample preparation

For STM measurements, a drop of the corresponding solutions of compounds **1** and **2** in 1-phenyloctane with appropriate concentrations was placed onto freshly cleaved highly oriented pyrolytic graphite (HOPG). The tunneling tip was then immersed directly into the droplet and the sample surface was imaged at room temperature. All images presented in this work were collected in constant current mode. The scales of the images have been calibrated using the visualized lattice of the underlying HOPG.

Lamellar Patterns of 1

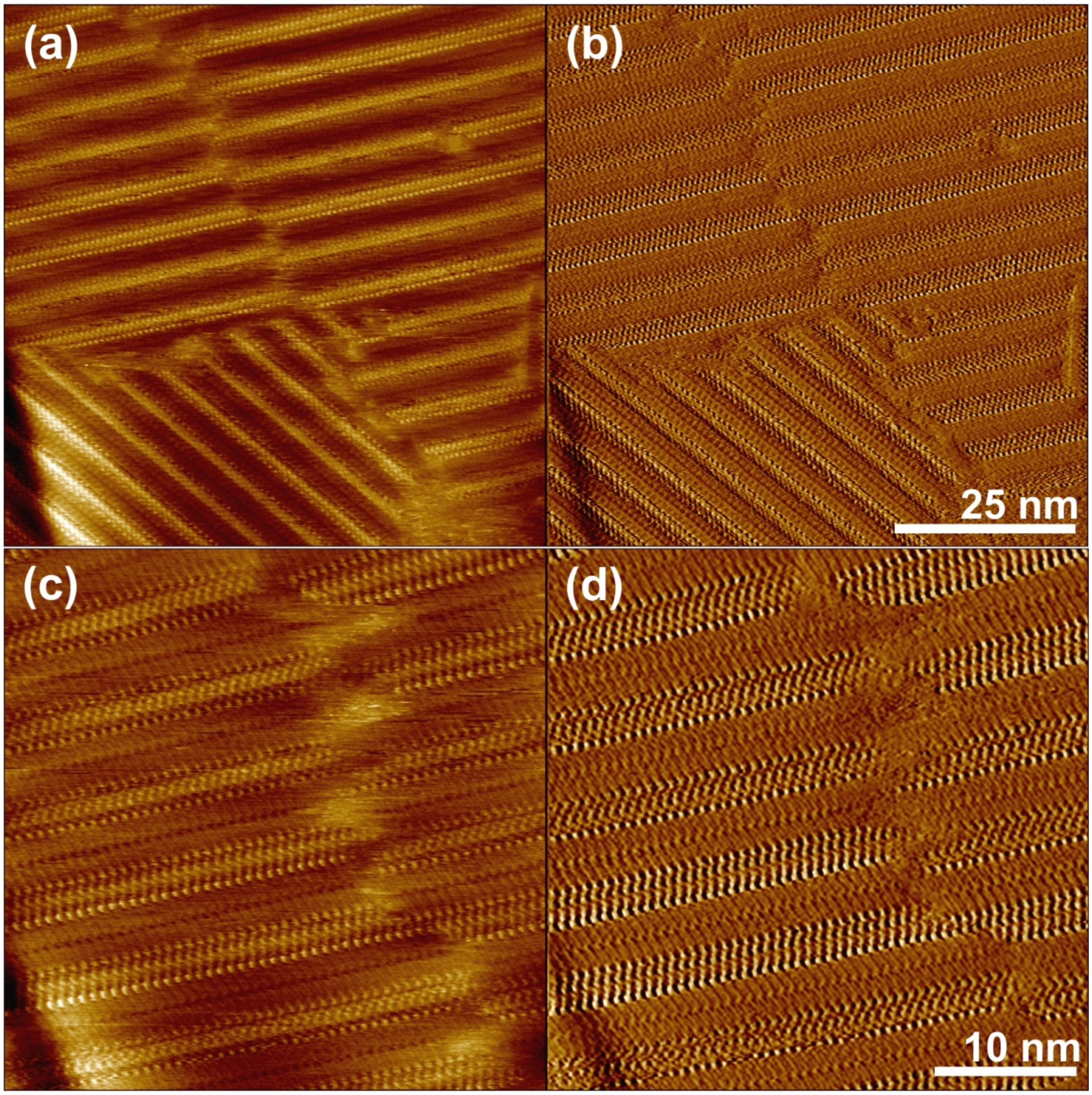

Figure S1. STM images of complex **1** (c = 5 x 10^{-4} M) revealing lamellar structures. (a & c) are height images and (b & c) are their respective current images

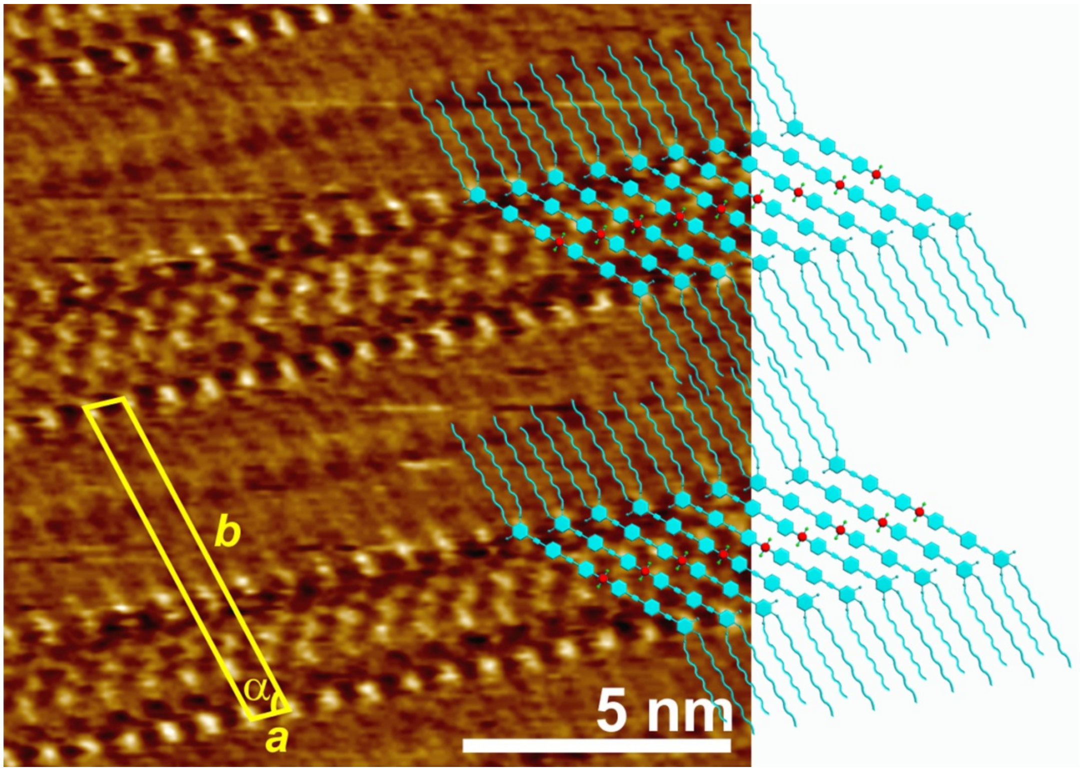

Figure S2. STM images of complex **1** (c = 5 x 10^{-4} M) and molecular packing within lamellar structures (enlarged Figure **1** in the main text).

Tiling patterns of 1

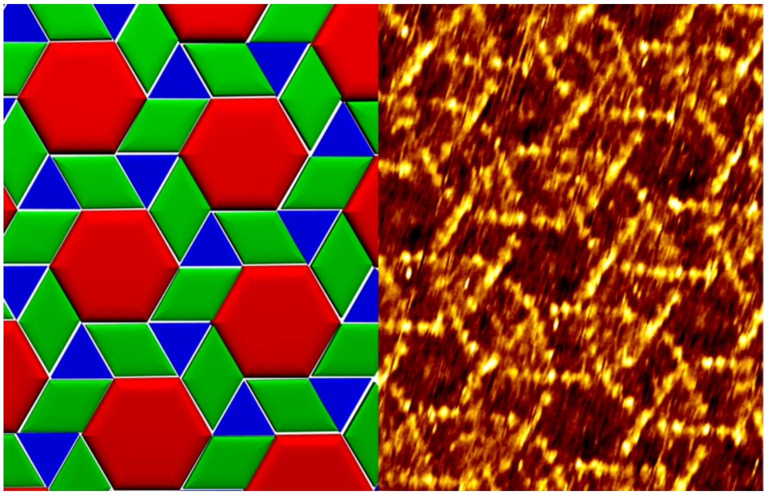

Figure S3. Left: surface tessellation showing the rhombitrihexagonal AT tiling. Right: STM height images of **1** at an HOPG/1-phenyloctane interface (1 × 10^{-6} M). Z scale is 0.2 nm. Tunnelling condition: V_{bias}= 690 mV, I_{set} = 8.0 pA.

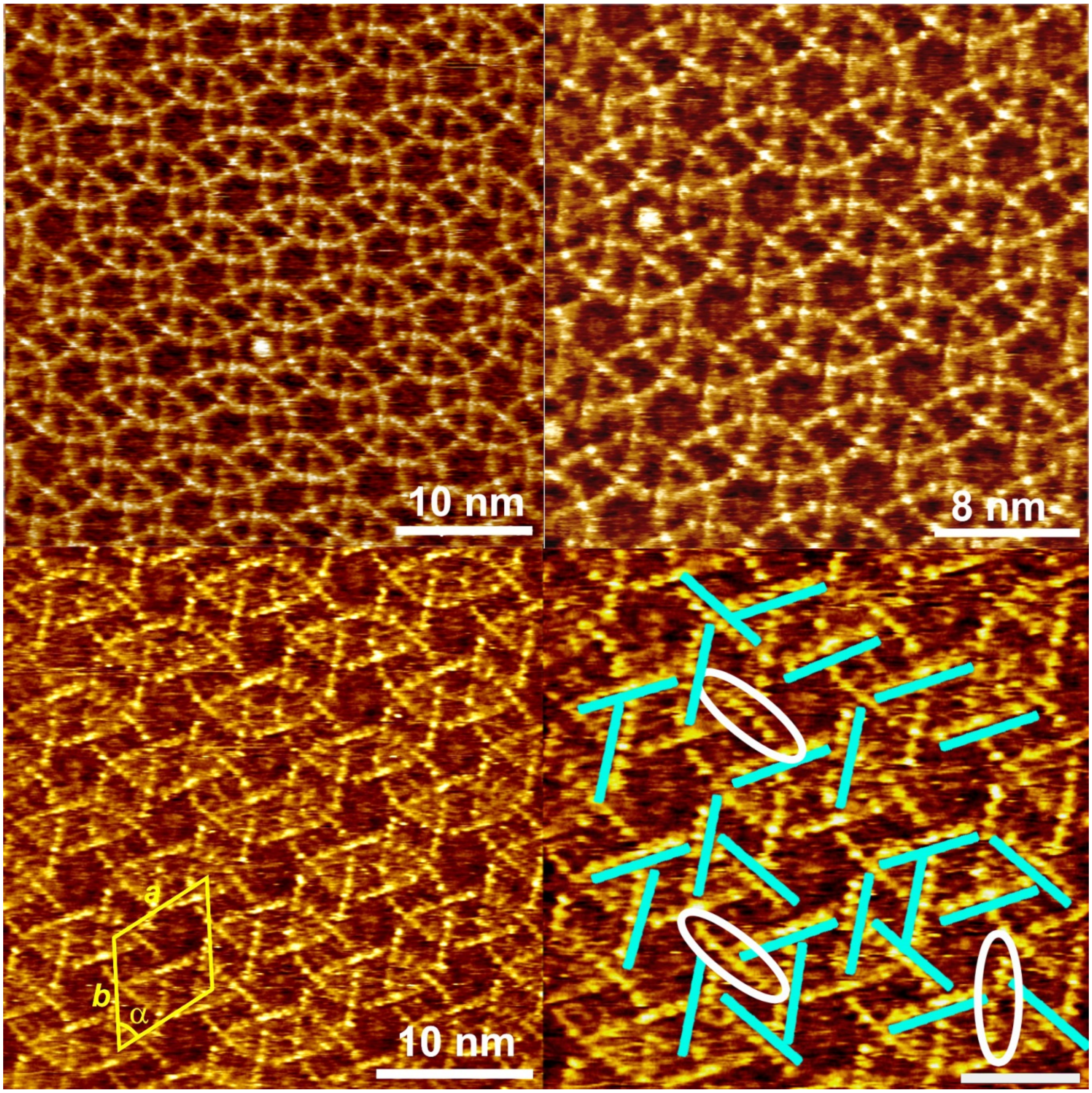

Figure S4. STM images of complex **1** (c = 1 x 10^{-6} M) revealing AT patterns at different magnifications. The blue segments have been added to identify straight molecules. Some slightly bent units can also be observed (see white circular shapes).

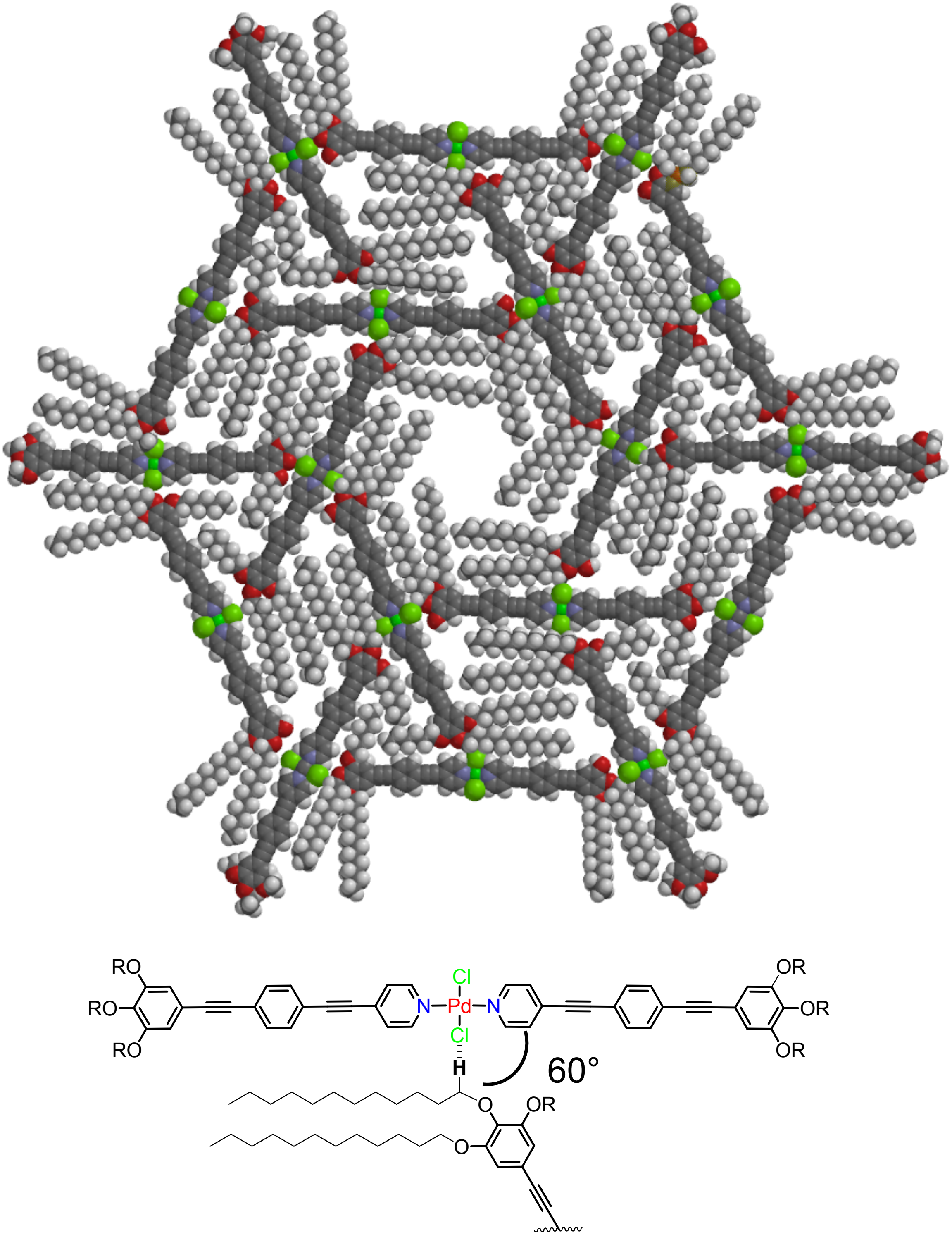

Figure S5. Proposed space-filling model (AM1 level, Spartan) of the AT tiling showing CH···Cl interactions involving the OCH_2 groups of the central alkyl chains and the Cl ligands.

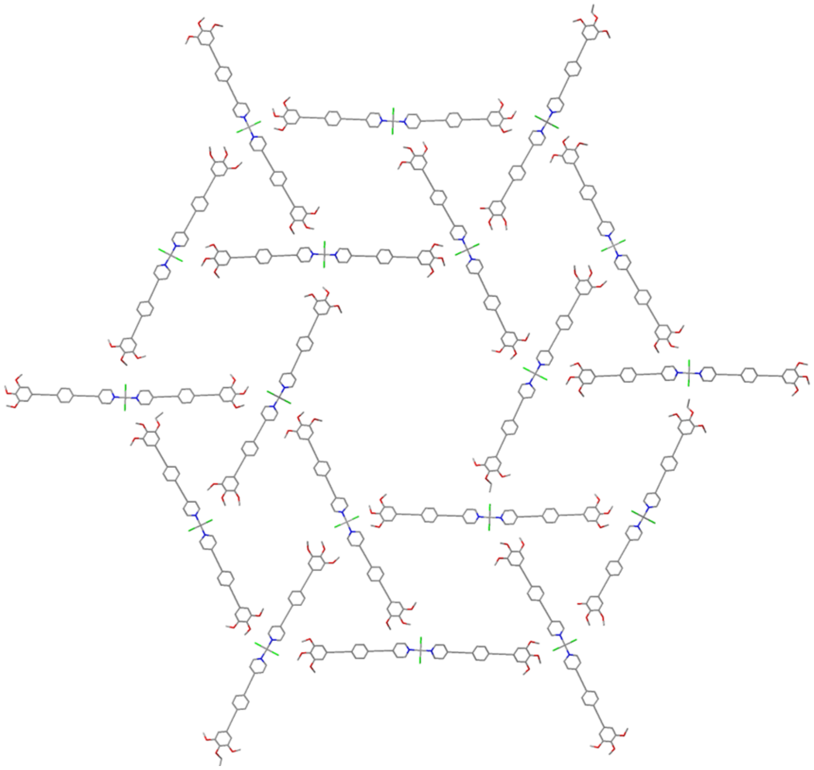

Figure S6. Alternative wireframe model of the AT tiling (chains have been removed for clarity) (AM1 level, Spartan).

Hybrid Patterns

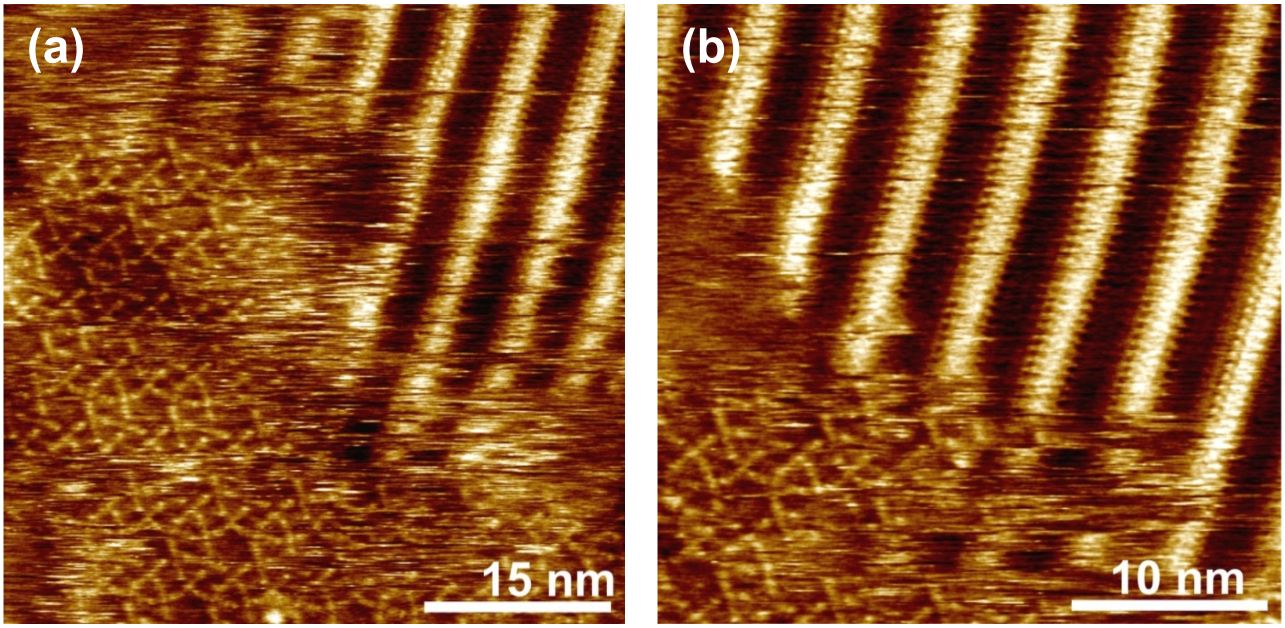

Figure S7. STM images of complex **1** showing both lamellar and AT patterns at intermediate concentration of 1 x 10^{-5} M

Grid morphology of 2

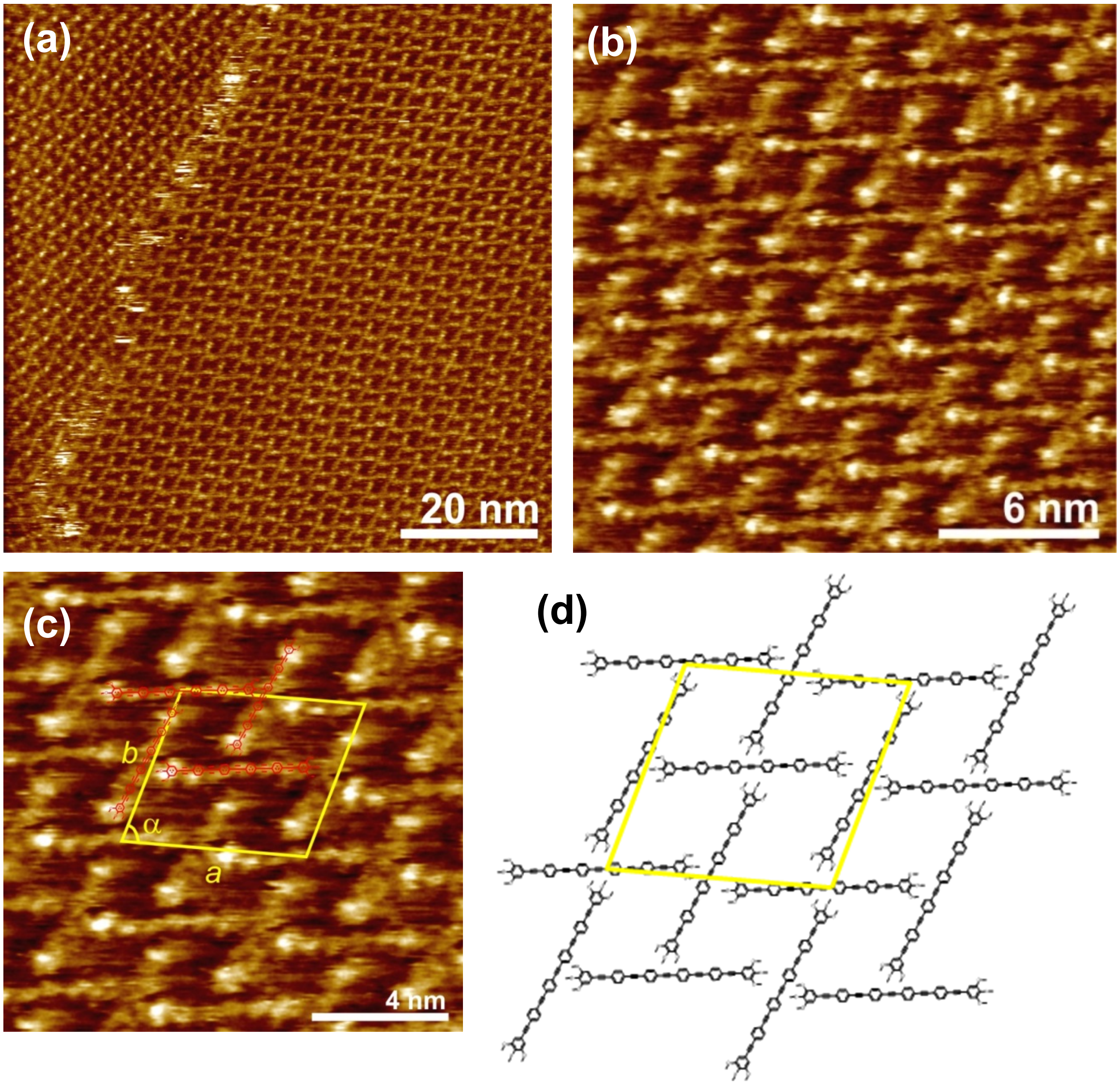

Figure S8. STM images of non-metallic system **2** (c = 1 x 10^{-6} M) showing uniform grid-like morphology (a) Two domains with similar patterns (b) magnified image (c) High-resolution height image revealing aromatic fringes partly overlaid by the chemical structures of **2** (in red) and the unit cell marked in yellow (d) Resemblance of the pattern with chemical structures.

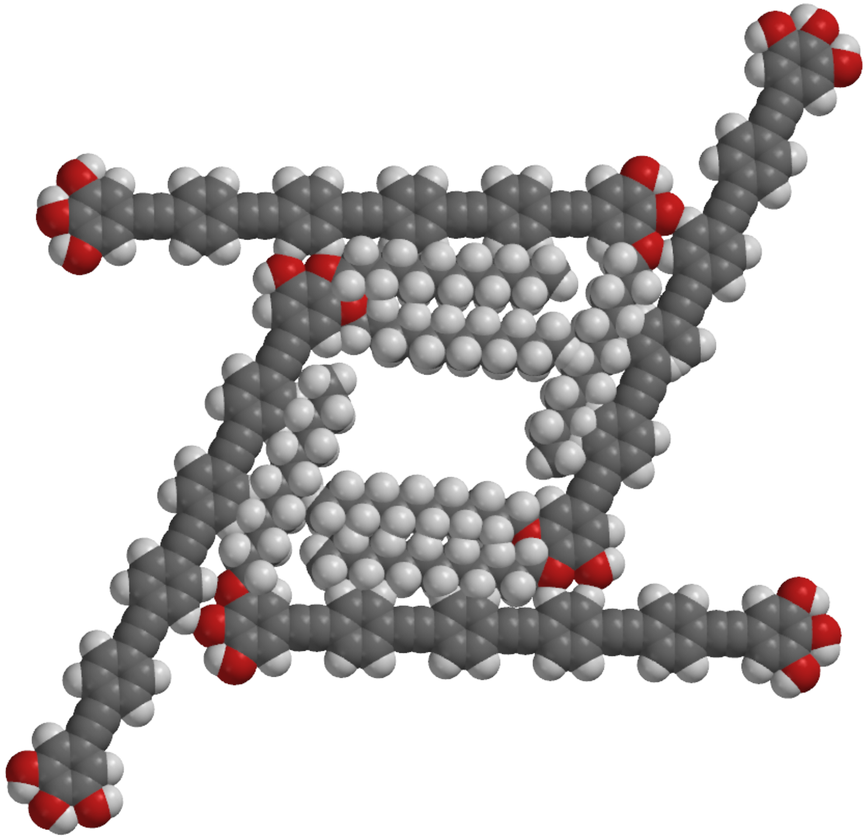

Figure S9. Proposed molecular packing of **2** within grid-like patterns in which all alkyl chains are on the HOPG surface showing C-H (aromatic)···O interactions.

Geometry-optimized Structures

S10 a)

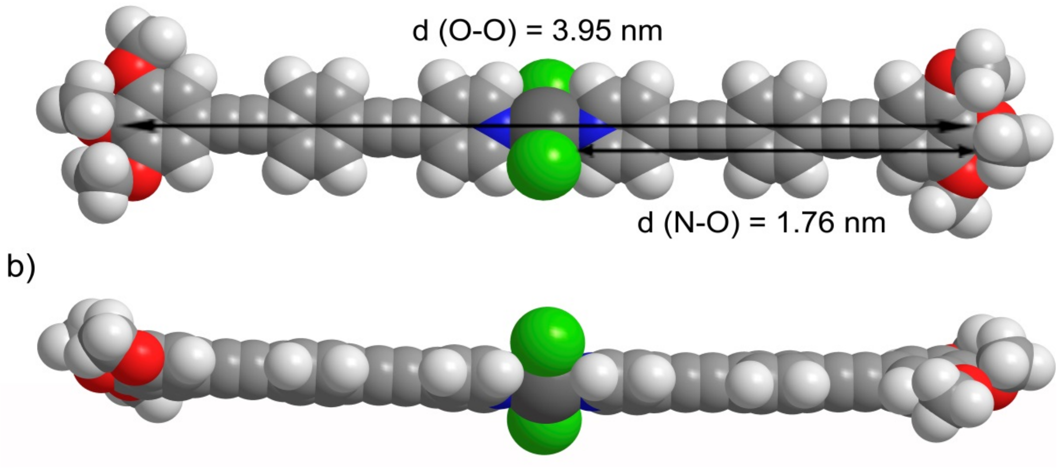

S11

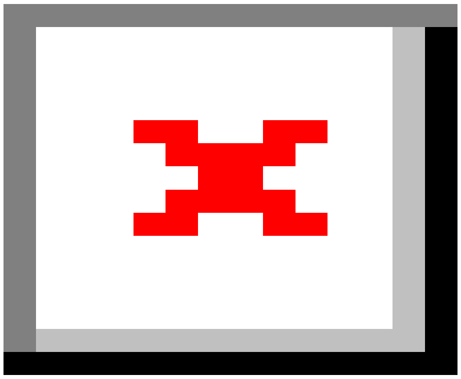

Figures S10 and **S11** are space-filling models of compounds **1** (dodecyl chains replaced by methyl groups) and **2** respectively (with axial and perpendicular views). The estimated molecular dimensions were determined by molecular mechanics (MMFF94 force field, Conjugated Gradients). Color code: Carbons: Grey; Oxygens: Red; Nitrogens: Blue; Hydrogens: White; Chlorines: Green
